# End‐user preferences for pounded yam and implications for food product profile development

**DOI:** 10.1111/ijfs.14770

**Published:** 2020-09-01

**Authors:** Bolanle Otegbayo, Tessy Madu, Oluyinka Oroniran, Ugo Chijioke, Olabisi Fawehinmi, Benjamin Okoye, Abiola Tanimola, Patrick Adebola, Jude Obidiegwu

**Affiliations:** ^1^ Department of Food Science and Technology Bowen University Iwo 232101 Nigeria; ^2^ National Root Crop Research Institute Umudike 440110 Nigeria; ^3^ International institute of Tropical Agriculture Ibadan 200132 Nigeria

**Keywords:** Appearance, end‐user preferences, food product profile, Nigeria, pounded yam, quality trait, region, textural quality gender

## Abstract

Pounded yam is a popular food in Nigeria. This study reports end‐user preferences for pounded yam and implications for trait evaluation by breeding programme. The study was carried out in two pounded yam‐consuming regions in Nigeria: south‐east and south‐west. Multistage sampling technique was used to collect information from users along food chain. This involved market, individual, key informant interviews and focus group discussions. Responses of participants were used to develop product profile of pounded yam from raw material (yam) to final product. Key user‐preferred quality traits for pounded yam in both regions were colour and textural quality followed by taste and aroma which are lesser attributes. There were regional differences in ranking of these quality attributes but no gender difference. This information will be useful in determining food quality indicators that can be used to select breeding lines for preferred quality traits in pounded yam.

## Graphical abstract

The study reports end user preferences for pounded yam in two pounded yam consuming regions (south‐west and south‐east) in Nigeria and its implications for trait evaluation by breeding programme. Key user‐preferred quality traits for pounded yam were colour and textural quality followed by taste and aroma. There were regional differences in ranking of these quality attributes.

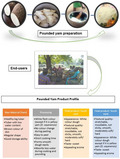

## Introduction

Yams are starchy tubers produced by about 600 known *Dioscorea* species (Obidiegwu & Akpabio, 2017). Nigeria alone, at 47.59 million MT, accounts for 65.5% of global production (FAO, 2018). Of the eight principal cultivated species in west Africa, white or Guinea yam (*D*.* rotundata* poir) and water yam (*D*.* alata*) are the most important. Yam is a major and preferred staple food for over 300 million people in west Africa (Alabi *et al*., 2019). It provides significant nutritional benefits in the form of calories from carbohydrates, protein, dietary fibre and micronutrients (Asiedu & Sartie, 2010; Apara, 2013).

Yam is intimately linked with the socio‐cultural life of the people. It serves as a special component of traditional gifts, fines and also an important ritual object for petitions and appeasement to the gods. The yearly New Yam Festivals in many parts of the country (Imo, Bayelsa, Ekiti, Osun, Ebonyi, Enugu, Anambra, Delta, Rivers, Niger) are glamorous traditional and social events that have gained international recognition and had lifted the status of the crop (IITA, 2004; Obidiegwu & Akpabio (2017).

**Figure 1:**
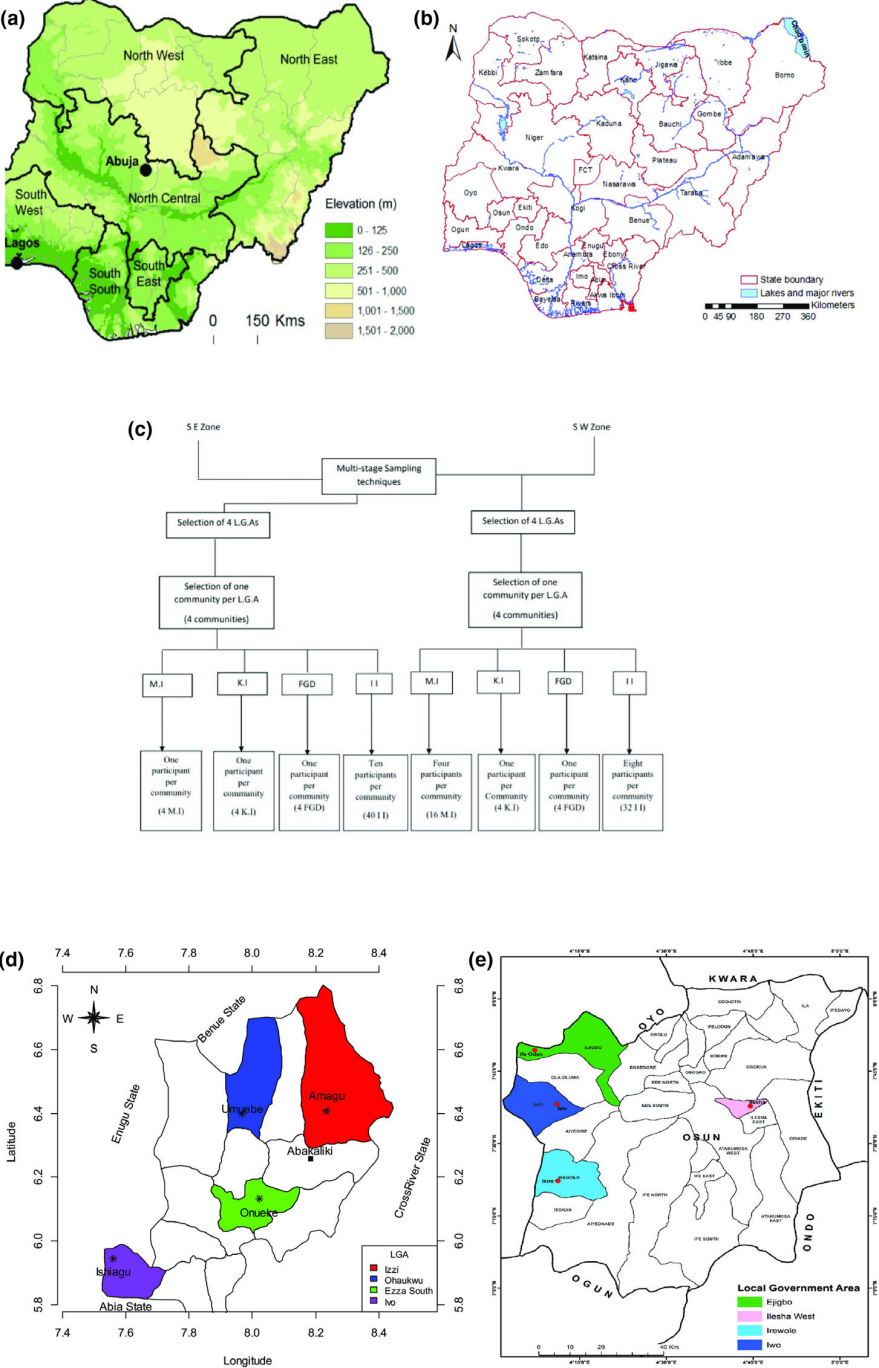
(a) Map of Nigeria showing geopolitical zones where yam is produced and consumed. Source: Okorie *et al*. (2013). (b) Map of Nigeria showing states where the study was carried out. Source: Ekpo *et al*. (2013). (c) Schematic representation of the survey method. (d) Areas in which survey was carried out in south‐east (Ebonyi State) region of Nigeria. (e) Areas in which survey was carried out in south‐west (Osun State) region of Nigeria.

Pounded yam is a glutinous dough prepared by peeling, boiling, pounding and kneading yam tubers (Otegbayo *et al*., 2005). Nweke *et al*. (2013) referred to pounded yam as the ‘ultimate food’ in status among yam consumers in Nigeria. In Nigeria pounded yam is commonly consumed majorly in the following geopolitical zones: south‐west; Ondo, Ekiti, Osun and Oyo States, south‐east; Abia, Anambra, Delta, Edo, Enugu, Ebonyi and north central; Tiv and Idoma areas of Benue State, Niger State and north‐east; Taraba State (Fig. 1a).

Traditionally, the tuber is peeled, boiled for 15–20 minutes, then pounded and kneaded to get a glutinous dough. Cold water may be sprinkled on the dough, pounded again before adding hot or cold water to soften it to the desired consistency (Otegbayo, 2018). The pounding process takes about 20 minutes. Previous authors (Konan *et al*., 2003; Otegbayo *et al*., 2005; Nindjin *et al*., 2007; Akissoe *et al*., 2009, 2011; Otegbayo et al., 2017) reported that textural quality is the most important food quality attribute considered by consumers of pounded yam. Textural quality derives from the structural elements of the pounded yam and can be measured by deformation, disintegration, and flow under a force (Bourne, 2002). Texture conveys set of complex sensory properties, important for pounded yam by consumers, including stretchability, smoothness. adhesiveness (stickiness) and cohesiveness (mouldability) (Egesi *et al*., 2003, Otegbayo *et al*., 2005; Nindjin *et al*., 2007; Akissoe *et al*., 2009; Otegbayo et al., 2017).

Our study focused on defining the key user‐preferred quality traits for pounded yam. These traits will be linked to biophysical and functional properties of yam, which will in turn lead to development of high‐throughput phenotyping protocols for rapid screening of user‐preferred quality traits in breeding programmes.

## Materials and methods

### Study area

The study was carried out in two yam‐producing and pounded yam‐consuming regions in Nigeria Ebonyi State in the south‐east and Osun State in the south‐west (Fig 1b). Ebonyi State shares boundaries on the north with Benue State, to the west with Enugu State, to the east by Cross River State and to the south by Imo and Abia State. Farming is the predominant occupation of the people in this state, majority of who are small‐holder farmers. Osun State is an inland state in south‐western Nigeria with boundaries on the west with Oyo State, Ondo and Ekiti States in the east, Kwara State in the north and Ogun State in the south.

These two states were chosen because of their intense yam production and consumption. Bergh *et al*. (2012) classified these states as ‘high yam yield’ States. The two states belong to two different geopolitical zones in Nigeria (south‐west and south‐east); hence, they were also selected to determine if there are differences in preferred food quality attributes of pounded yam produced in the two regions. The major crops produced in these states include yam, cassava, rice, maize, cocoyam, cowpea and tomatoes and are also involved in animal production.

### Survey method

In both regions, we adopted a multi‐stage sampling technique described by Forsythe *et al*. (2021). (The flow chart of the survey method is presented in Fig. 1c). Stage one involved purposive selection of LGAs in the state based on high levels of yam production and pounded yam consumption (Fig. 1d,e). Stage 2 comprised selection of one community from each of the selected local government areas. Stage three involved random selection of respondents in the selected communities. In Ebonyi State, the first stage involved purposive selection of local government from the three Agricultural Zones. These comprised of Ivo LGA in Ebonyi south, Izzi and Ohaukwu in Ebonyi north, and Onueke in Ebonyi central. These LGAs were selected based on intensity of yam production and consumption. In the second stage, one community each was also purposively selected: Onueke in Ezza south LGA, Amagu in Izzi LGA, Umuebe in Ohaukwu LGA and Ishiagu in Ivo LGA. The selection of the communities is based on large concentration of yam farming activities in these communities. In stage three, the participants for FGDs were randomly selected from the list of farmers in the study area collected from Agricultural Development Programme (ADP). Eight FGDs comprising of four male and four female groups in each community were conducted, forty participants for individual interviews (II) (ten in each community), four participants for market interviews(MI) in each community and four key informants (KI), making a total of 108 respondents in the south‐east region.

In Osun state, stage one was the purposive selection of four local governments comprising of Iwo LGA, Ejigbo LGA, Irewole LGA, and Ilesha LGA based on the fact that these local governments are famous for yam production in the state. The second stage is the random selection of one community from each of the selected local government areas: Iwo in Iwo LGA, Ife‐Odan in Ejigbo, Ikire in Irewole and Ilesha in Ilesha LGA. The communities were selected because of their intense yam production and pounded yam consumption Stage three is the random selection of respondents per communities who are either farmers, traders or processors and are conversant with eating of pounded yam. Five focus group discussion (FGDs) (three male and two female groups) were conducted, forty individual interviews (II) (ten from each community) were conducted, fourteen respondents were interviewed for market interview (MI), and five key informant (KI) interviews were conducted; thus, the total number of respondents in south‐west region was 110. In both regions, the total number of respondents was 218 (n = 218).

Ethical clearance was obtained from National Research Committee, and each of the participants gave their consents by signing the consent form prior to the interviews.

### Data collection and analysis

Qualitative data were collected from both primary and secondary sources. The primary data were collected using question guides designed by Forsythe *et al*. (2021) to determine the key preferred quality attributes in pounded yam from both regions. Local informants helped in facilitating the interviews interaction and interpretation in local languages, pidgin and English language. Relevant information, such as household structure, production, utilisation, varieties cultivated, their preferred varieties, the processing method for pounded yam in both regions (if there are differences in the processing methods and what are the differences), quality attributes preferred in good quality pounded yam and to know if there are gendered differences in the responses to the questions asked especially relating to the key preferred quality attributes. In the focus group discussions, copious discussions were encouraged and the participants were allowed to give testimonies, myths and proverbs attached to the consumption and key preferred quality traits in pounded yam. Responses from the participants were then transcribed to pivot table on Microsoft excel for thematic coding. The data obtained were analysed using simple descriptive statistics such as mean, percentages, charts mean and standard deviation.

## Results and discussion

### Socio‐economic characteristics of pounded yam end‐users

**Figure 2:**
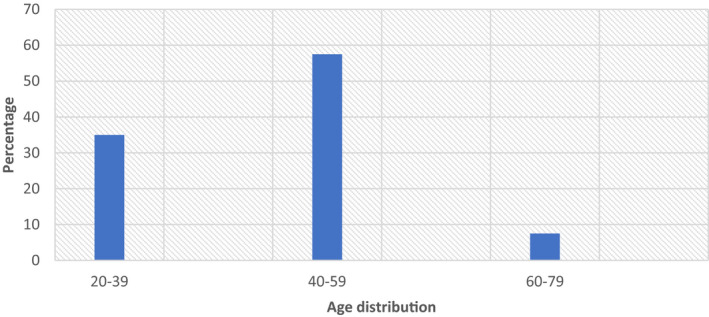
Age distribution across all questionnaire respondents.

The age distribution of the respondents (Fig. 2) showed that majority (69.6%) of the respondents were between the ages of 40 and 60 years with a mean age of 50.39 ± 8.98 while those between 20 and 39 years were about 35% and 60% and above constitute 7.5% of the respondents. This implied that a greater percentage of the respondents were in their active age. This is in agreement with the report of Izekor & Olumese (2010) and Otegbayo *et al*. (2010) who reported the average active years of yam farmers in SE and SW Nigeria as 51–60 years. They will therefore need food that is high in calories like pounded yam in order to have strength for the strenuous physical activities that they are more likely to be engaged in.

**Figure 3:**
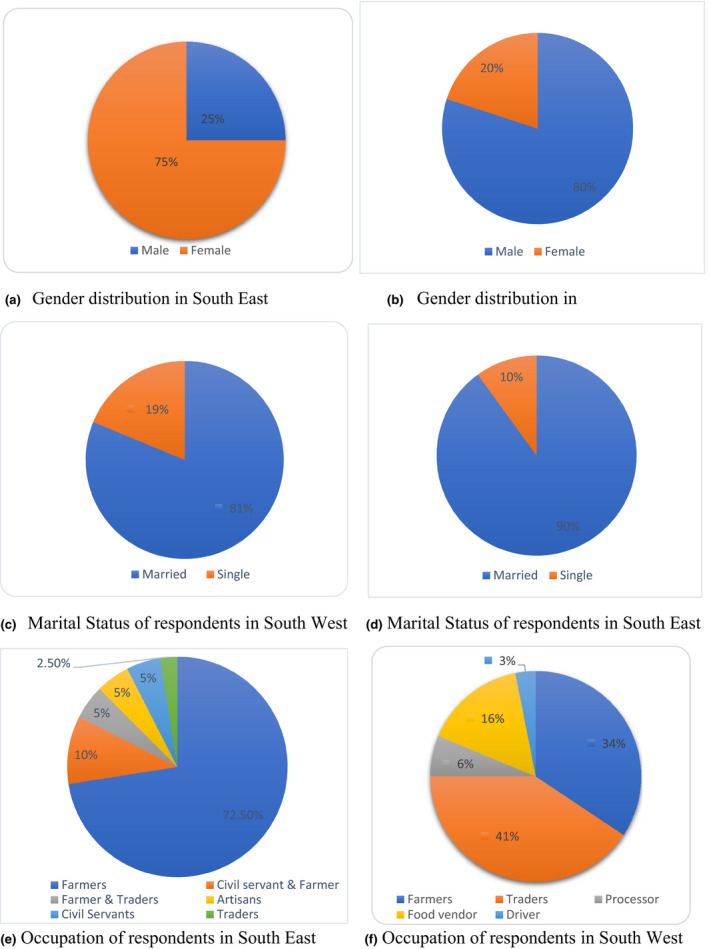
Socio‐economic characteristics of respondents.

In terms of gender, in the south‐east the majority of respondents were males (80%) (Fig. 3a), while most of the respondents in the south‐west were females (70%) (Fig. 3b). The dominance of male respondents in the SE corroborates the saying that yam is a *male* crop (Korieh, 2007; Bergh *et al*., 2012) in this region. The implication of having more females that participated in the SW is in agreement with Regina *et al*. (2011) that male and female participation in yam activities vary based on regions and that there is currently shift in gender roles with more women now involved in yam planting, trading and adoption of new technologies in yam production such as yam minisett technologies. In addition, having more women participation in SW may positively influence their preference for choice of yam variety which can be used for pounded yam since women are the homemakers and the onus lies on them to satisfy the food demand of their families.

In terms of the household structure of the participants, in the SE, 75% of the participants are heads of their households. This was followed by 17.5% who are wives to the household heads. About 2.5% of the participants are sons and daughters to the household heads, respectively, while those who are single constitutes 2.5% of the participants. Husbands thereby have a strong influence on the food that is consumed at the household level in this region. In SW, 28% of the participants are the heads of their households, 62.6% are wives, while 9.4% are either sons or daughters in the household.

Farmers’ interview showed that they have a local method of measuring their yam production quantity. In SE, it is measured in terms of ‘chains’, where twenty‐five chains are equivalent to one hectare. 25% of the farmers reported that their yam production capacity is *seventy‐five chains* (that is they plant 5 hectares of yam), while the production scale of the remaining 75% is about *twenty‐five chains* (1 hectare). The production scale in SW is measured in terms of ‘number of heaps’, and this depends also on the size of heaps, which can be ‘*karugbe*’ (big heaps) or ‘*ebe were*’ (small heaps). Moreover, *karugbe* is the most common among the respondents. 87.5% of farmers in SW region are small‐scale farmers, known as ‘*alada kekere’*, cultivating about 3000 heaps (according to the farmers, this is equivalent to one acre), while 12.5% are large‐scale farmers (*alada nla*), cultivating above 10,000 heaps. Hence, most of the farmer respondents in both regions are small‐scale farmers.

In both SW and SE, the majority of the respondents were married (81% and 90%, respectively) (Fig. 3c,d). In terms of the occupation, majority of respondents from SE region were farmers (72.5%) and others were artisans, civil servants, traders and farmers, who combined trading with farming and civil servants who also farm. In the SW, about 40.6% of the respondents were primarily traders which is closely followed by farming (34.4%), and then other vocations such as processors, drivers and food vendors (Fig. 3e,f). Being primarily yam traders and yam farmers their professions will help them to be able to identify yams that will give good quality pounded yam.

### Pounded yam preparation

**Figure 4:**
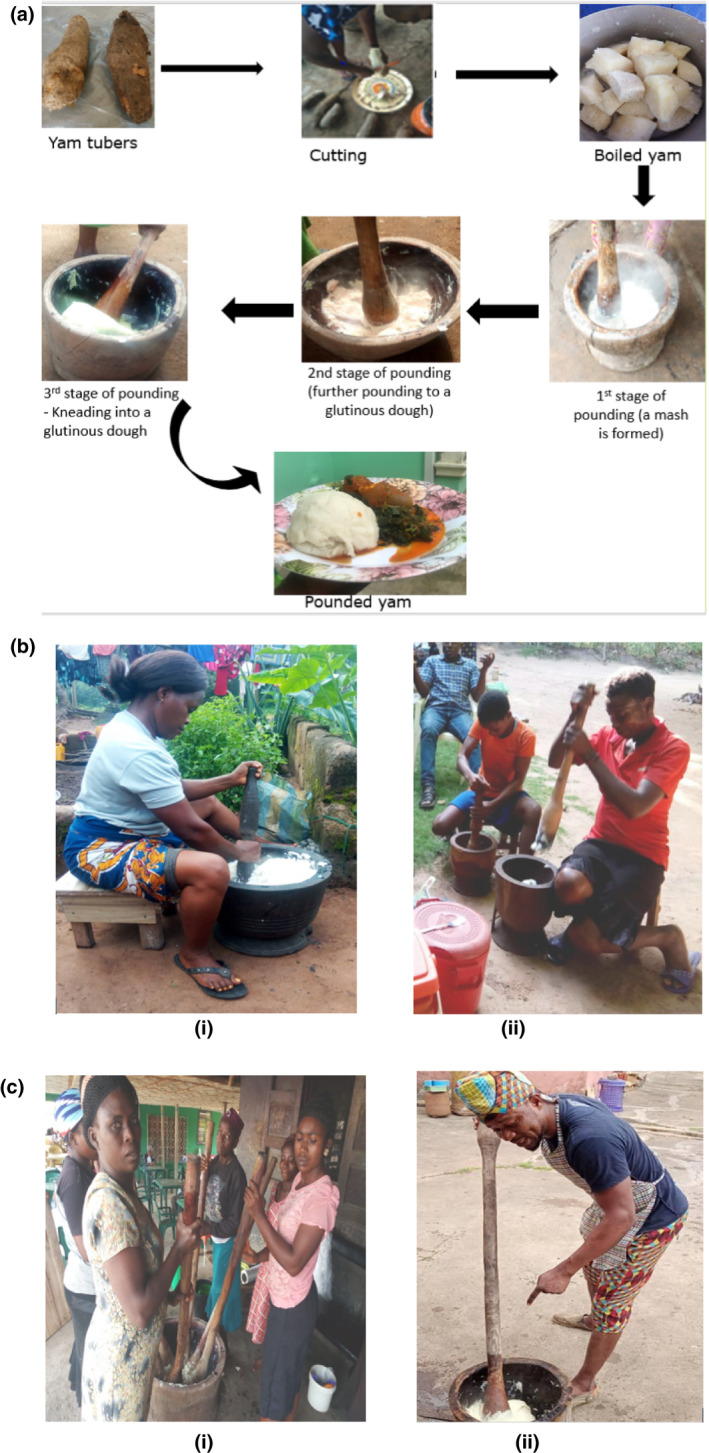
(a) Process of producing pounded yam. (b) (i and ii): Yam pounding techniques in the south‐east region. (c) (i and ii): Yam pounding techniques in the south‐west region.

Based on the responses from participants in both regions, the pounded yam processing method follows the same procedure (Fig. 4a). The yam tubers are peeled, sliced or cut into small chunks, washed, cooked for about 20–30 mins, and then pounded. However, it was observed that the pounding technique in the two regions was slightly different. In the SE region, the pounding pestle is shorter and the pounder may sit down (Fig. 4b) whereas the pestles are usually longer in the SW region and the pounding is done while standing, and two or more people can be pounding in a big mortar simultaneously (Fig. 4c).

### Species and varieties of yams cultivated

The yam varieties cultivated by the participants were all landraces from *Dioscorea rotundata and D*. *alata* species. No improved or recently released variety was mentioned by the farmers during the interview sessions. The varieties cultivated in the SW region were: *Lasinrin, Gbongi, Aro, Amula, Awana, Gambari, Efuru, Totoro, Tankalubo, Keso, Ewura eweseni, Gbongi‐kamilu, Ofegi, Atoja* and *Aga*. From the survey, the respondents were asked to rank the cultivated varieties in terms of their preference for making pounded yam. The preferences of the yam varieties for good quality pounded yam varied among the communities. In Ife‐odan in the SW, Lasinrin was ranked as the most preferred variety, *Gbongi* was the most preferred in Iwo, Aro in Ilesha and Awana in Gbongan. Lasinrin ranked second in Iwo community while it was rated the same along with *Efuru* and *Gambari* by the Gbogan community. Generally, the order of preference (from most to less preferred on a scale of 1–5 where 1 represents most preferred and 5 least preferred) for pounded yam in the SW is as follows: L*asinrin*> *Awana*> *Aro*> *Amula*, *Odo* (Table 1).

**Table 1:** List of cultivated, preferred and non‐preferred yam varieties for pounded yam and order of ranking for making good quality pounded yam

	List of cultivated varieties*	List of preferred varieties for good quality pounded yam	Order of ranking for making good quality pounded yam				List of non‐preferred varieties for good quality pounded yam
			Female		Male		
			Variety	Rank	Variety	Rank	
South‐west	Amula, Aro, Awana, Efuru. Gbongi, Efuru, Lasinrin. Odo, Gambari Ewura eweseni, Totoro, Keso, Tankalubo, Gbongi Kamilu, Ofegi, Aga, Atoja	Amula, Aro, Awana, Efuru. Gbongi, Efuru, Lasinrin. Odo, Gambari	Lasinrin Awana Gbongi, Aro Efuru, Odo, Amula	*1* *2* *3* *4*	Lasinrin Gbongi	*1* *2*	Ewura eweseni, Totoro, Keso, Tankalubo, Gbongi Kamilu, Ofegi, Aga, Atoja
South‐east	Oko, Igum(Nwaopoko), Obiaoturugo, Iboki Mvula, ojir, onicha, ifara, okpabe, Abi Orunte Ji igwe, Abana, obela, amage	Igum, Abi, onisha obiaoturugo, Orunte, Izzamgbo	Igum Abi Obiaoturugo Onicha Orunte	*1* *2* *3* *4* *5*	Igum Abi	1 2	Oko, mvulaokpabe, iboki Abana, igweobela, ifaraamage

* Figures in italics are the ranks in order of preference of varieties for making pounded yam based on simple ranking method where 1 represents the most preferred variety and 5 represent least preferred variety for good quality pounded yam.

**Figure 5:**
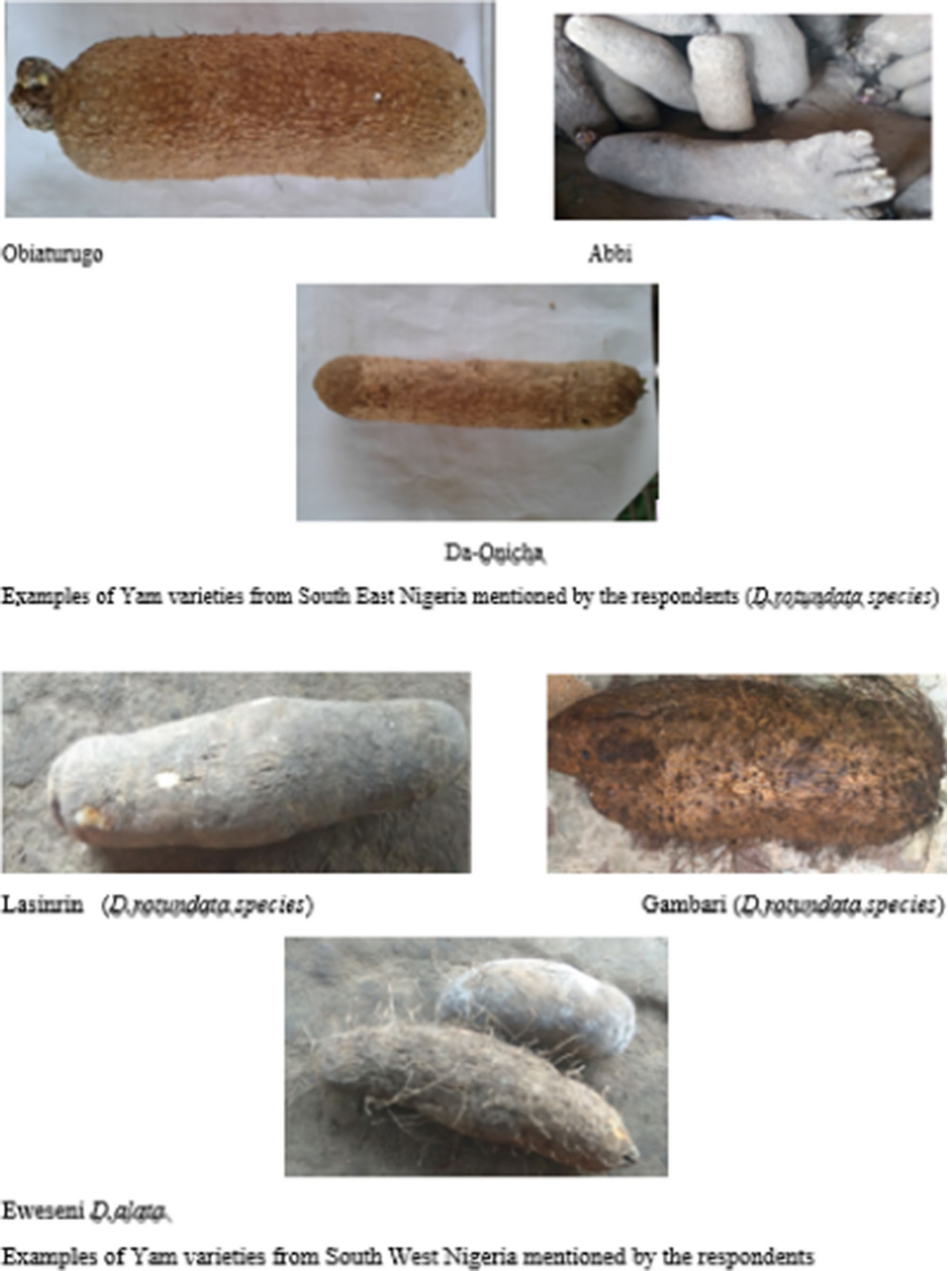
Pictures of some yam varieties cultivated by respondents.

In the SE, the following varieties were cultivated: *Oko, Igum Nwaopoko, obiaoturugo, Iboki Mvula, ojir, onicha, ifara, okpabe, Abi, Orunte, igwe, Abana, obela, and amage*. *Igum* was the most preferred variety for pounded yam followed by *abbi*, then *Obiaturugo*. The least preferred variety for pounded yam was *orunte*. The order of preference of the variety for pounded yam in this region (based on a scale of 1–5) was *Igum*> *Abbi*>*Obiaturugo*> *Onicha*> *Orunte*. Table 1 shows the list of some yam varieties and their preferences for good quality pounded yam by gender and region. Table 2 and Fig. 5 describe the characteristics of some of the varieties mentioned.

**Table 2:** Some yam varieties cultivated by the respondents and their characteristics

Specie	Variety	Characteristics
*D*. *rotundata*	Obiaturugo	Late maturing yam and a high yielding yam. tolerates pests and diseases. It is white fleshed, smooth skin, cylindrical shape, less hairy. Good for pounded yam., boiled yam and fried yam. It is easy to pound and very stretchable, has good taste, aroma and it is good for many things including pounding, boiling and frying.
	Abbi	Early maturing yam. It is moderately resistant to virus. it does not have high yield. The tuber irregular in shape, although not rotundus but tapering. The head region is usually bitter which extends towards the bottom with a reducing intensity. It gives stretchable pounded yam but with a lot of lumps It is good for frying.
	Da‐onicha	Early maturing, multiple tubering, high yielding, white fleshed tuber and smooth skin. Long cylindrical tuber. Good for pounded yam, boiled, fried and roasted yam.
	Lasinrin	Early maturing yam, it is high yielding and pest resistant, it could be yellow or white flesh. It is very good for pounding, boiling and frying. The pounded yam is very stretchable.
	Gambari	Early maturing yam, long round, sturdy and heavy. The flesh is white. It is high yielding and tolerates pests and diseases. It gives very mealy boiled yam and white stretchable pounded yam.
	Olodo	Late maturing yam, high yield has high moisture content when fresh, white fleshed, round in shape. The pounded is usually too soft and the boiled yam is soggy when fresh but after yam storage for about 3 months, the pounded yam becomes good.
*D*. *alata*	Eweseni	High yielding, High water content, pounded yam does not form a dough easily, not smooth and it could be very fibrous or waxy when boiled.

### Characteristics that influence consumer preferences of yam for making pounded yam

**Figure 6:**
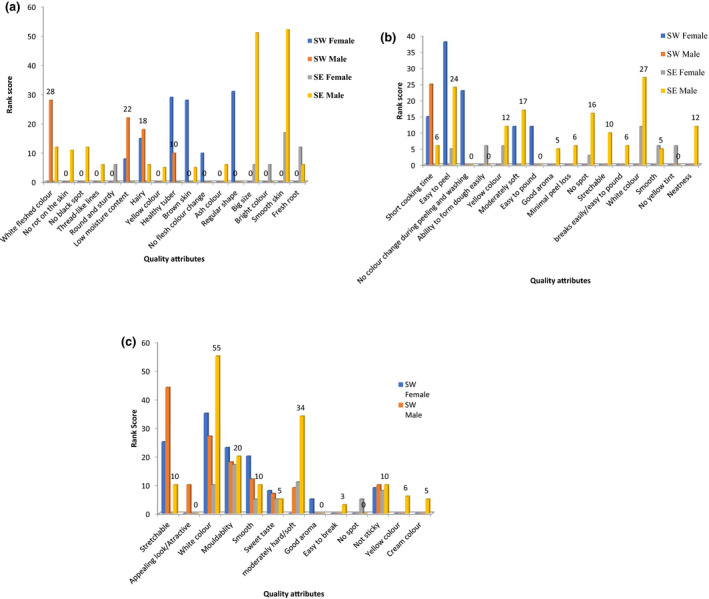
(a) Preferred traits at harvest. (b) Preferred traits during processing. (c) Preferred traits of final product.

The respondents were asked questions on what are the preferred quality traits in pounded yam? There were numerous traits that the respondents identified from planting of the yam to the final product (pounded yam) (Fig. 6a–c); however, these traits were similar in both regions. From this, it was observed that there were three characteristics that are important in consumers’ preference for pounded yam. They are (i) Agronomic and postharvest, (ii) processing, and (iii) cooked/ready to eat final products. These characteristics are discussed below.

#### Agronomic/postharvest characteristics

The agronomic/post‐harvest characteristics that influenced participants’ preferences for variety of yam for pounded yam were similar in both regions. The participants reported that while the yam is growing (agronomic characteristics) the yam should have big vines, dense canopy with lush green dense leaves and be pest resistant. The post‐harvest characteristics they preferred were as follows: big healthy heavy tubers (the yam should be as heavy as stone when you carry it or like a small log of wood they said the yam to be pounded should be very big like tree stem), with low water content (they detect this by pinching the skin of the tuber). If the flesh brings out water rapidly, they do not like it, because it is an indication that the tuber will have high water content. In SW, this is depicted by a local vocabulary ‘*ko gbodo sunkun’* (it must not shed tears). The next important characteristics were brown colour of the skin. Respondents said, ‘the skin of a good yam should be brown in colour and mature when you touch it’. This was followed by smoothness. The participants posited that the skin of yam for good quality pounded yam should be smooth without spots or rot on the body. In addition to these characteristics, the female participants also preferred the early maturing varieties. It was observed that most of the preferred varieties from SW were actually the early maturing varieties. These are summarised in Table 3.

**Table 3:** Agronomic/post‐harvest characteristics of yam tubers influencing survey participants’ variety preferences in south‐east and south‐west Nigeria

Socio‐economic factors	Preferred quality characteristics (agronomic/postharvest)^*^	Characteristics of yam that give less preferred quality of pounded yam
Region: South‐east and south‐west Nigeria	Pre‐harvest: Fat vine, Green dense leaves, Big heavy tubers (heavy like stone or log of wood) and healthy tubers (without spots or disease or rot) Post‐harvest: Low water content (when scratched with the finger it must not bring out plenty water (‘*ko gbudo sunkun*’ Colour of the skin must be brown (this is the sign of a matured tuber) White yam flesh colour when peeled/scratched Smooth skin/soft hair (without spot or rot) Pest resistant Early maturity (especially women) High Storability	Level of yam tuber freshness (indicated by holes, insect infestation, rotten yam), High water content tiny tubers, poor yield, lots of deep‐rooted hair on the flesh of the tuber when peeled Flesh colour change when peeled Unappealing look; tiny tubers, shrunk tubers or woody tubers.
By gender:		
Female	Early maturity, Regular shape, Big size Fresh healthy tubers, Soft hair on skin, No flesh colour change when peeling	Flesh colour change when peeling, high water content, big size of irregular shape because it is not easy to peel
Male	Low water content White flesh colour, Soft hair on the skin	Hard, high water content, flesh colour change when peeling, unappealing look (shrink, rotten, irregular shape)

#### Processing characteristics

From the results of the survey, the processing characteristics preferred in varieties of yam that participants use for pounded yam from both regions SE and SW can be classified into two groups: physicochemical and technological characteristics of the tuber during processing. The most important processing characteristics were colour of the yam flesh (Table 4). The participants reported that flesh colour of the tuber through each processing stage from peeling to cooking is important. The colour must not change; it should be bright white or cream (except if it is yellow yam; *D*.* cayenensis*). Other characteristics were freshness/ healthy tuber, easy peeling and presence of deep root hair strands on the peeled tuber. The participants in both regions unanimously responded that good quality yam for pounded yam should not have any blemish as it impacts negatively on the end product.

**Table 4:** Preferred quality characteristics of yam during processing into pounded yam

Socio‐economic factors		Preferred quality characteristics during processing (physicochemical)				Preferred quality characteristics during processing (technological)			
		Peeling	Washing	Cooking/ boiling	Pounding	Peeling	Washing	Cooking/ boiling	Pounding
General		White flesh/ bright colour Tuber not too soft (must not contain too much water)	No colour change during washing. Washing water should be clear not milky and slimy	No colour change during cooking and should not absorb too much water	Easy to pound, no lumps, not sticking to the pestle and dough should swell during pounding	Yam skin should be easy to peel (no spikes or thorns)	Water used for washing the tuber should be clear and not milky and slimy. There should be no change in the colour of the yam flesh during washing	Yam should have short cooking time, and should not absorb too much water	Boiled yam should form dough easily during pounding.
Gender	Female	White flesh colour	Colour of flesh still remain white in water, water should be clear	Yam must not absorb too much water	Forms a dough easily	Easy to peel		Short cooking time and not absorb too much water	Easy breaking of boiled yam at early pounding; forms a smooth dough easily
	Male	No colour change in the flesh	Water should not be milky or too slimy	No colour change when cooked	Not lumpy			Short cooking time	Not lumpy

#### Characteristics of cooked/ready‐to‐eat final products

From the responses of participants from the SE region, pounded yam with appealing colour (white) was ranked first by both male and female participants. The participants believed that good quality pounded yam should have pure white colour. This makes it more appealing to the eye. The next factor in order of importance is textural quality (Table 5). They reiterated that a yam variety preferred for good pounded yam should give a dough that is stretchable, mouldable and smooth (without lumps). The preferred pounded yam must also have good taste and aroma, and these were ranked 3rd and 4th, respectively, by the participants (some of them said the pounded yam should be as sweet as honey). However from the SW, the participants stated that a yam variety used for pounded yam should form a glutinous dough which must be stretchable, not sticking to the fingers, smooth (not lumpy), mouldable, soft, white colour, with sweet taste and appealing aroma.

**Table 5:** Key user preferred sensory quality attributes in pounded yam by participants

	Region		Preferred quality characteristics	Less preferred quality characteristics
	South‐east	General	White dough, stretchable, smooth, mouldable, moderately soft, sweet taste, appealing smell easy to swallow, keeps long,	Dark colour, sticky dough, hard or too soft lumpy, does not stay long (retrogrades), bitter taste, unappealing smell
Gender		Female	White colour, appealing look mouldable, smooth, stretchable, sweet taste, good aroma,	Bitter taste, unappealing smell, dark unappealing dough colour, not mouldable, too soft, not stretchable, hard, lumpy
		Male	Appealing look ( white colour) stretchable, mouldable, smooth, sweet taste, soft	too soft, dark unappealing dough colour, lumpy, hard, bitter taste
	South‐west	General	Stretchable dough, mouldable, smooth, moderately soft, appealing colour appealing, pleasant taste and smell.	Dark colour, sticky dough, hard or too soft lumpy, does not stay long (retrogrades), bitter taste, unappealing smell
Gender		Female	Stretchable dough, mouldable, smooth, moderately soft, appealing colour appealing, pleasant taste and smell.	
		Male	Stretchable dough, mouldable, smooth, moderately soft, appealing colour appealing, pleasant taste and smell.	

The outcome of the pivot table analysis (Fig. 6c) further collaborates the most important characteristics that are desired in the raw materials (yams), during processing and in the final products. The result indicates that in the south‐west, the important characteristic that is desired among the male is white flesh colour with a score of 28 while the female gender group prefers yam with regular shape with a score of 31. In the south‐east region, the most important characteristic that both the males and females desire in the raw material is that the yam should have smooth skin with a score of 52 and 17, respectively.

During processing of the raw material, the most important attribute required by the males in the south‐west region is that the yam should have short cooking time with a score of 25, whereas the attribute desired of the female in the region is that it must be easy to peel with a score of 38. The most important characteristics in the south‐east region during processing is that the raw material (yam) should be white in colour for both the male and female gender groups with score of 27 and 12, respectively.

The most important attribute desired in the final product (pounded yam) among the male gender group in the south‐west is that the pounded yam should be stretchable with a score of 44 while the most preferred trait of the final product by the female is that it should be white in colour with a score of 35. The males in south‐east prefers pounded yam that is white in colour with a score of 55 while the most preferred attribute of the final product by the female is that it should be soft with a score of 11.

### Development of a food product profile

Based on the result of these surveys from the two regions, quality attributes expected in a good pounded yam can be grouped into two major factors: appearance and textural quality. We used a pairwise ranking test for the respondents to determine which of the two major quality attributes ranked first in preference in the two regions. In the SW, textural quality was ranked first followed by appearance/colour. The preference of the key preferred quality attributes in this region was as follows: textural quality> colour>taste> aroma. The textural attributes were ranked in the following order of preference: stretchability> mouldability> stickiness> smoothness> moderately hard/soft. In the SE, the result of the pair wise ranking showed that colour/appearance was ranked first, followed by textural quality. However, the ranking of the textural quality attributes was in the same order as those from the SW. The ranking of colour/appearance as the most important quality attribute of pounded yam in the SE was further reinforced by their local proverb which states that ‘*Anya na ebuzo eri tupu onu erie’* (‘The eyes will eat first before the mouth’). The ranking of textural quality first rather than colour in pounded yam is not unexpected for participants from the SW since they have other staple food products from Yam (‘amala’) which is also highly appreciated but it is brown in colour and other staples such as ‘lafun’ (grey or cream) and wheat (brown), which are coloured; hence, they are used to eating coloured products. Thus, culturally, textural quality plays an important role in their food quality preferences. Taste and aroma ranked lower as other less important organoleptic factors in pounded yam. Taste and aroma might have been minor preferred attributes in pounded yam in both regions because it is usually consumed with soups, which may mask the taste and aroma of the pounded yam. The gender difference in the preferred attributes was in the agronomic factors, where the women preferred early maturing yam varieties and colour of the pounded yam (SW females) but the key quality preferred traits in pounded yam are the same.

From the results of the survey, the food quality attributes preferred in pounded yam by the participants can be grouped into three: Quality attributes preferred from raw material (yam tubers), quality attributes preferred during processing of pounded yam, and food quality attributes preferred in the final product‐pounded yam (Table 6). Thus, the preferred food product profile may be described as illustrated in Table 6. The implication of this food product profile to breeders is that consumers will prefer varieties of yam that produce big and healthy tubers, have low water content, and have lower tendency to oxidative browning (no change in colour throughout the processing steps).

**Table 6:** Product profile of preferred/ good quality pounded yam

Product profile*
Raw material (tubers)
Healthy big tuber
Tuber with low water content
Brown colour of skin
Regular shape
Processing
White flesh colour (except if it is yellow yam (*D*. *cayenensis*)
Easy to peel
No colour change during peeling
Short cooking time
Absorbs less water during cooking and pounding
Forms smooth dough easily
Final product
South‐east
Appearance: White colour dough
Textural quality: Stretchable, mouldable, not sticky, smooth, moderately soft/ hard
Taste: sweet
Appealing aroma
South‐west
Textural quality: Stretchable, mouldable, not sticky, smooth, moderately soft/ hard
Appearance: White Colour dough except if it is yellow yam (*D*. *cayenensis*)
Taste: sweet
Appealing aroma

* The preferred traits are in order of preference.

## Conclusions

Based on the result of this study, key preferred food quality attributes in pounded yam were appearance and textural quality. However, there were regional differences in the ranking of these food quality attributes. In the SE region, colour ranked first followed by textural quality while in the SW, textural quality ranked first followed by colour. However, the textural quality attributes preferred in both regions were the same and followed the same order: stretchability> mouldability> stickiness> smoothness> moderately hard/soft.

Food culture was also observed to play a dominant role in these food quality preferences. Hence from this study, the key user‐preferred quality traits for pounded yam are appearance and textural quality followed by taste and aroma. In future studies, the preferred traits should be linked with biophysical and functional properties of yam tuber in order to provide breeders with criteria for high throughput screening. For instance polyphenol oxidase enzyme activity in the yam, tuber can be analysed to determine if it can predict the colour of pounded yam produced from the tubers. Starch properties (swelling, amylose) or chemical composition (protein, carbohydrates, starch, non‐starchy) can be studied as possible determinants and/or predictors of any of the key‐user preferred quality traits. Such intrinsic factors in the tubers can then be used for rapid screening of user‐preferred quality traits in variety development and deployment programmes. It is also recommended that key preferred attributes in pounded yam may be studied in more yam‐producing and yam‐consuming regions in Nigeria.

## Author Contribution

**B. Otegbayo:** Conceptualization (supporting); Funding acquisition (supporting); Investigation (lead); Methodology (supporting); Project administration (lead); Supervision (lead); Writing‐original draft (lead); Writing‐review & editing (lead). **Tessy Madu:** Conceptualization (supporting); Investigation (lead); Methodology (supporting); Writing‐original draft (supporting); Writing‐review & editing (supporting). **Oluyinka Oroniran:** Investigation (supporting); Methodology (supporting); Project administration (supporting); Supervision (supporting). **Ugo Chijioke:** Investigation (supporting); Methodology (supporting); Project administration (supporting); Supervision (supporting). **Olabisi Fawehinmi:** Investigation (supporting); Methodology (supporting); Supervision (supporting); Writing‐original draft (supporting). **Benjamin Okoye:** Formal analysis (lead); Investigation (supporting); Methodology (supporting); Writing‐review & editing (supporting). **Abiola Tanimola:** Investigation (supporting); Methodology (supporting); Writing‐review & editing (supporting). **patrick Adebola:** Writing‐original draft (equal). **Jude Obidiegwu:** Investigation (supporting); Methodology (supporting); Writing‐original draft (supporting).

## Conflict of interest

The authors declare no conflict of interest in this work.

## Ethical guidelines

This study was approved by the National Research Ethics Committee. Research teams obtained ethical approval prior to the fieldwork. Participants were informed about the study, they could stop the interview at any point, written consent from sensory panellists and from consumers participating in this study was obtained, and the research respected the rules of voluntary participation and anonymity.

### Peer review

The peer review history for this article is available at https://publons.com/publon/10.1111/ijfs.14770.

## Data Availability

Research data for this study can be available on request from the corresponding author. The authors are not sharing the data publicly due to privacy or ethical restrictions.
